# What else can we do to mitigate contamination of fresh produce by foodborne pathogens?

**DOI:** 10.1111/1751-7915.12231

**Published:** 2014-12-27

**Authors:** Shlomo Sela (Saldinger), Shulamit Manulis-Sasson

**Affiliations:** 1 Department of Food Quality and Safety, Institute for Postharvest and Food SciencesBet Dagan, Israel; 2 Department of Plant Pathology and Weed Research, Agricultural Research Organization (ARO), The Volcani CenterBet-Dagan, Israel

The beginning of the 21st century and the third millennium marks a revolution in science and technology with innovations in multiple areas, including space science, computer sciences, communication, biotechnology, nanotechnology and the human genome project, to name a few. Yet at the same time people are still getting ill from eating contaminated food, with a heavy toll on public health and economy. This is not merely the case in developing countries with poor sanitation but also in rich and developed countries. In fact, during the past few decades, increasing number of outbreaks of foodborne illnesses associated with raw or minimally processed fruits and vegetables in Europe and the USA was reported. This parallels the expansion in the consumption of raw and partially processed fresh produce, including ready-to-eat (RTE) salads, which are recommended by doctors and dieticians as nutritious and healthy food.

Unlike foods of animal origin, such as meat, poultry and dairy products, which can undergo a thermal process (e.g. cooking, pasteurization, etc.) in order to inactivate human pathogens, fresh produce cannot undergo such processes because they induce physiological damages and deteriorate the organoleptic properties of these products. In order to ensure the safety of partially processed RTE foods, chemical and physical decontamination treatments were developed. The most common one includes washing with tap water and dipping in disinfectant solutions, such as hypochlorite (Goodburn and Wallace, 2013). However, unlike the thermal processes which can achieve the ‘gold standard’ 5-log reduction in the numbers of the challenged pathogen of choice in food of animal origin, this goal cannot be met with the current industrial treatments in fresh produce (Goodburn and Wallace, 2013). This is quite surprising as foodborne pathogens, such as *Escherichia coli*, nontyphoidal *Salmonella*, *Campylobacter* and *Listeria monocytogenes*, are all sensitive to the disinfectants used by the industry, at the relevant concentrations and exposure time. The lack of an efficient kill-step forces the fresh produce industry to largely rely on preventive measures, such as good agricultural practice (GAP) and hazard analysis of critical control points (HACCP).

There are several plausible explanations for the limitation of disinfectants to completely eradicate foodborne pathogens on fresh produce. Two major explanations are (i) the localization of bacteria in protected niches, either in cracks and crevices on the plant/fruit surface, or within internal organs following internalization, which prevent direct contact with the disinfectant; and (ii) formation of biofilms, or integration of foodborne pathogens into existing biofilms on the plant's surfaces, which hampers the ability of the antimicrobial agent to kill the pathogen.

Contamination of fresh produce may occur during each step of the food chain from farm to fork, yet because postharvest treatments cannot be relied upon to eliminate pathogens, prevention of preharvest contamination on farm seems to be the most important step in reducing human health risk (Beuchat, 2006). Sources of human pathogens on the farm include low-quality irrigation water, application of contaminated organic fertilizers, such as untreated or partially treated animal manure, as well as close proximity to livestock operations. Preharvest contamination may result in the introduction of pathogens into the processing plant, establishment of biofilms on food-contact surfaces and subsequent cross-contamination of lots to be distributed at a national or international scale, leading to outbreak.

Current contamination routes of fresh produce in the field include surface contamination by splashes of irrigated water, rain or during floods, and internal contamination via root internalization, natural openings such as stomata or trichomes, as well as via physical or biological damage to the plant organ (Critzer and Doyle, 2010).

It is commonly accepted that bacteria residing on the surface of plant are exposed to environmental stresses, such as irradiation, desiccation and lack of nutrients, which limit bacterial survival. On the other hand, internal plant's tissues provide bacteria with a favourable environment, rich in nutrients and water, and protected from external stresses. Likewise, surface-attached bacteria will be more prone to bactericidal activity of external disinfectants used during industrial processing, while internally localized bacteria will be protected and might even proliferate under the appropriate conditions.

A common approach for hazard mitigation is the multiple hurdle approach. In this context, we would like to discuss here the introduction of additional hurdles, through agro-technology, which might be applied in future years to limit the contamination of plant in the field, thereby enhancing the safety of partially processed and RTE fresh produce. This can be achieved by adapting known agro-technologies previously utilized for plant protection against diseases also for minimizing plant contamination by foodborne pathogens. These include (i) development of breeding programmes towards cultivars/varieties that are less prone to colonization by foodborne pathogens, (ii) grafting as a mean to limit migration of foodborne pathogens from the rhizosphere to the phyllosphere, (iii) induced resistance against foodborne pathogens and (iv) application of antagonistic microorganisms to limit survival of foodborne pathogens in the plant environment.

## Breeding programmes for enhancing food safety

A vast amount of biodiversity exists in plants, which is utilized successfully by farmers, seed companies and researchers for decades as a mean to develop new cultivars/varieties with favourable traits, such as increased productivity, quality and resistance to diseases. Similarly, induction of mutation and the emergence of genetic engineering in plants have resulted in the advent of new cultivars with specific quality and disease resistance traits, which had a dramatic impact on the economy, globally. Although productivity, quality and plant protection remain the major incentive for screening and breeding programmes, it is envisaged that food safety will push the research also towards harnessing these tools for the generation of crops resistant to colonization by foodborne pathogens. This idea is supported by studies demonstrating dependency between colonization and plant's cultivar. For example, colonization of *Salmonella* in tomato (Barak *et al*., 2011) and of *E. coli* O157:H7 in spinach leaves (Mitra *et al*., 2009) was found to be affected by the nature of the cultivar. High variation in stomata internalization by *Salmonella* was also observed among different cultivars of iceberg lettuce grown in the field under identical agricultural setting (S. Sela, unpubl. data). Thus, a combination of screening for colonization-resistant plants' lines, accompanied by directed breeding programmes that would take into account safety-related traits, should potentially contribute to the ongoing battle against contamination of fresh produce by human pathogens.

## Grafted plant as a novel approach to limit bacterial internalization through the root system

Internalization of plants through the root system is considered a possible contamination route by foodborne pathogens (Critzer and Doyle, 2010). *Salmonella* and *E. coli* strains were reported to enter through the root system of several plants, including lettuce, spinach and corn, yet root internalization seems to be cultivar-specific. Grafting susceptible scion onto internalization-resistant rootstock might be a promising alternative to limit entry of foodborne pathogens into vegetables. This idea was proved to be useful for inducing resistance against soil-borne diseases (King *et al*., 2008), as well as for minimizing the penetration of toxic contaminants from the soil into the plant (Otani and Seike, 2007). We envisage that such a technique, in vegetable crops, where grafting is possible, may prove a useful approach for generating additional hurdle against transport of foodborne pathogens from the rhizosphere to the phyllosphere.

## Stimulation of the plant's immune response

Accumulating evidence has led to the understanding that enteric bacterial pathogens are not merely contaminants of plants but can also adopt an endophytic lifestyle, using the plant as an alternative host (Holden *et al*., 2009; Schikora *et al*., 2012). Similar to plant's pathogens, foodborne pathogens are specifically sensed by the innate immune system of the host plant, which restricts their colonization and survival. The plant immune system is induced upon exposure to pathogen-associated molecular patterns (PAMPs) and initiate systemic defence response (PAMP-triggered immunity; PTI) in order to limit the dissemination of the pathogen in the plant. Recent data demonstrate the capability of some human enteric pathogens to induce plant's immune response (Holden *et al*., 2009). Interestingly, colonization of *Salmonella* type 3 secretion system (T3SS) mutants on plant was found to be compromised, suggesting that human pathogens are utilizing T3SS effectors to suppress the plant immune response (Schikora *et al*., 2012). Based on these findings and on similar studies, it can be speculated that stimulation of the plant's defence system may be utilized as a novel agro-technology to limit colonization of plants by foodborne pathogens. Indeed, exposure of *Nicotiana benthamiana* to the *Salmonella* flg22 peptide has induced PTI and limited the multiplication of the pathogen on the leaves (Meng *et al*., 2013). These findings support the notion that elicitation of the plant immune response may act as an additional measure to limit plant contamination by foodborne pathogens. This approach certainly requires further studies to assess its effectiveness using different immune system elicitors, vegetable crops and human pathogens.

## Antagonistic microorganisms

The rhizosphere and the phyllosphere are rich with microorganisms that reside in close proximity to the plant tissues and have intimate interactions with the plant. It has been long known that microorganisms have multiple beneficial properties on plants, including increased crop productivity and resistance against multiple plant diseases by serving as biocontrol agents. Microorganisms secrete a multitude of metabolites, which might alter the physical and biochemical properties of the plant environment. For example, Bacteriocins, which are ecologically important family of metabolites, are produced by some bacteria to facilitate their colonization of specific niche by inhibiting or killing antagonistic microorganisms. Indeed, studies have demonstrated the presence of antagonistic microorganisms in the rhizosphere and phyllosphere of fresh produce and their potential utilization as safeguard against human pathogens (Goodburn and Wallace, 2013). The distribution of microorganisms depends on many variables, including the plant species, soil type, fertilizers, water, geographic region and climate, to name a few. The search for antagonistic microorganisms from the rhizosphere and phyllosphere of vegetable crops in combination with high-throughput screening programmes has the potential to identify antagonistic microorganisms with the ability to limit colonization of plant by foodborne pathogens.

In summary, we envisage that during the next years, modern agriculture will adopt an integrated food safety management system as part of a multiple hurdle approach to limit fresh produce contamination and protect public health. Safety of fresh produce should be strictly kept at all stages of the food chain from the farm to the consumer. In this essay, we have tried to point out on a number of potential, not necessarily new, agro-technologies that in combination with other approaches, such as GAP and HACCP, may be utilized to limit on-farm contamination of fresh produce. These include the use of natural biodiversity to screen for vegetables' cultivars resistant to foodborne pathogens, the generation of resistant plants via classical breeding or by genetic engineering, grafting technique to limit pathogens' migration from the rhizosphere to the phyllosphere, stimulation of the plant's immune response to restrict bacterial colonization, and the utilization of antagonistic microorganisms.
